# The experiences of family members of persons with intellectual disabilities who used residential care homes during the COVID-19 pandemic

**DOI:** 10.1080/17482631.2023.2288100

**Published:** 2023-12-06

**Authors:** Montserrat Puig Llobet, Montserrat Roca Roger, Teresa Nicolàs Silva, Gemma Pérez Gimenez, Zaida Agüera, Maria-Teresa Lluch Canut, Juan Francisco Roldan Merino, Carmen Moreno Arroyo, Marta Prats Arimon, Maria Aurelia Sánchez Ortega, Xavier Domènech Mascaró, Miguel Angel Hidalgo Blanco, Antonio Moreno Poyato

**Affiliations:** a Department of Public Health, Mental Health and Maternal-Child Nursing, School of Nursing, University of Barcelona, Health Sciences Campus Bellvitge, L’Hospitalet de Llobregat, Barcelona, Spain; b La Vinyota. Mollet Health Foundation, Mollet, Barcelona, Spain; c Head of Sick Medical Nursing Area and alternatives to hospitalization, Granollers General Hospital, Granollers, Barcelona, Spain; d Department of Mental Health, Campus Docent Sant Joan de Déu-Fundació Privada, University of Barcelona, Barcelona, Spain; e Department of Fundamental and Medical-Surgical Nursing, School of Nursing, Faculty of Medicine and Health Sciences, University of Barcelona (UB), L’Hospitalet de Llobregat, Spain; f Nursing and Occupational Therapy School (EUIT), Universitat Autònoma de Barcelona, Terrassa, Barcelona, Spain; g Personal attention to citizens. Upper Penedès Territorial Pediatric Care Team Upper Penedès-Garraf-Baix Llobregat Nord Primary Care Service. Vilafranca del Penedès, Barcelona, Spain

**Keywords:** Qualitative research, COVID-19, family, intellectual and developmental disabilities (IDD), Experiencies

## Abstract

**Background:**

The global COVID-19 pandemic has shown the vulnerability of some population groups, including persons with intellectual and developmental disabilities (IDD).

**Aim:**

The present paper will provide more clarity and understanding of the experiences of family members of persons with IDD housed in residential facilities in Catalonia within the period of maximum restrictions during the COVID-19 pandemic.

**Methods and procedures:**

Semi-structured interviews were conducted using an interpretive phenomenological qualitative approach. Study participants consisted of 14 relatives of IDD individuals who were institutionalized in residence facilities or homes. The guiding questions emerged from group discussions with relatives of those with IDD who did not participate in the subsequent interviews. Drawing from this group, the factors that were identified to have had the greatest impact on their lives were later used to guide the interviews. Data collection was carried out in face-to-face individual interviews that were recorded together with the observations of two researchers between February and October 2022.

**Results:**

Our analysis identified 4 main themes that developed into additional factors: the decision to stay at home or in the residence, fear, illness, and protocol. Individuals with IDD lost their daily routines, suffered from social isolation, and did not understand the situation.

**Conclusion:**

The results of this study allow for a better understanding of the experiences of families of persons with IDD in residential centres during the lockdown by identifying their needs and how to better support them in the future.

**Outcomes and results:**

Knowledge and understanding of these events should allow for better management of similar situations in the future.

## The impact of the present research

This study contributes to a growing body of research that examines the experiences of IDD individuals and their families during the early months of the COVID-19 pandemic. A qualitative phenomenological interpretative approach was used following the recommendations of qualitative research practices in health care. Our findings reveal that relatives of persons with IDD who lived in residential care homes felt that the residents were not adequately cared for by the staff, who applied measures for containment and contagion prevention that were disproportionate, resulting in their needs not being considered; these recommended measures had been developed for elderly care homes. Individuals with IDD lost their daily routines, suffered from social isolation, and did not understand the situation. The present findings show that residential care homes for persons with IDD require specific action protocols in crisis situations that are adapted to the needs and characteristics of each centre,thus adopting a model of care based on human rights.

## 1. Introduction

Persons with intellectual and developmental disabilities (IDD) present limitations in important areas of life, including language, mobility, learning, self-care, and independent living.

Some persons experience severe multiple disability with serious limitations requiring 24-hour support for many aspects of their lives: eating, drinking, grooming, and dressing, among others. Some also present behavioural disorders. In other cases, these individuals require only temporary support in certain aspects to lead a normal life. Thus, there are a wide range of different situations. In Spain, almost 300,000 people live with intellectual disabilities, although the data is not always very reliable due to poor reporting on the characteristics and degree of disability. When adults with IDD cannot be cared for by their family members, they are cared for in institutions subsidized by the public social services system, the majority of which are managed by third sector organizations. The responsibility and guardianship of these persons remains with family members, when they exist.

Due to the lack of resources to care for adults with IDD in Spain, most of these persons become independent from their families when they are over 30 years old and remain in the residential care homes until their death.

This lack of resources can sometimes mean that the location where they are cared for is far from their relatives’ place of residence.

It is estimated that in Spain there are 17,000 residential places for people with IDD distributed throughout approximately 1,000 centres.

In the present work, the study participants were relatives (parents and siblings) of persons with IDD who had very little autonomy to manage the basic activities of daily life and no capacity to participate in the activities that are institutionalized in residential care homes, where professionals provide 24-hour care. They could be visited by their family and friends whenever they wished and go on recreational outings, but they did not participate in any type of productive activity. These types of establishments vary in terms of capacity, ranging between 20 and 100 residents.

The relatives of IDD individuals who lived in supervised group homes of between 4 and 10 people also participated. These persons were able to carry out basic and instrumental activities of daily living with the supervision of another person and, when not living in the family home, they also attended work centres supported by care workers. Since group home accommodation was only needed after work and on weekends, if they wished, support from professional care workers was limited.

The global COVID-19 pandemic has shown the vulnerability of certain population groups, one of them being people with IDD (Tummers et al., 2020; WHO, 2020). They are a group that is vulnerable to both mental and physical health issues, including infectious disease (Cuypers et al., 2020). The lack of inclusivity shown in response to the pandemic has exacerbated its impact on the lives of persons with IDD and highlights how underdeveloped the current model of care is in terms of human rights. The situation has been particularly acute for those who are institutionalized. Since the beginning of the pandemic, they have been categorized as a “vulnerable population group” in Spain (Ministry of Health , 2019, Ministerio de Sanidad 2019), by assimilating them to institutionalized elderly people, with the official morbidity and mortality data presented for both groups together. From March 10 to 9 May 2020, excess mortality for people over 75 years of age in Spain increased by 75% compared to expected cases and was 65% of the general population. The non-governmental organization P*lena Inclusion* has estimated that around 100 people died in residential centres for people with intellectual disabilities during the pandemic and around 1,000 fell ill with COVID-19 (Europapress, 2020). The disparity in morbidity and mortality did not imply a differentiation in the standards dictated by health authorities. The same protocols were dictated for all institutionalized people.

On 20 March 2020, with the advance of the pandemic and the increase in mortality in nursing homes, the health authorities decided to close to the public all centres for both the elderly and those caring for people with IDD (residential care homes and group homes with care worker support).

Two days before this closure, family members who were legal guardians (parents or siblings) were asked if they wanted their institutionalized relatives to return home or continue in centres under lockdown, considering that visits would not be allowed in the latter until the health authorities allowed it. The decision was made solely by family members in the case of care homes, however, for those who lived in group home apartments with support, the clients were also included in the decision making.

Next, the residents of these centres were separated into isolated groups and facilities were compartmentalized so that no spaces were shared among groups. When one resident tested positive for COVID-19, the group members had to isolate themselves in their rooms for a minimum of 15 days or until the test showed negative. At first, direct contact with the infected person was also isolated.

This regulation was relatively easy to implement in the care homes, but almost impossible in supervised group homes due to lack of space, being standard apartments with bedrooms that were often shared and a living room for socializing.

Another regulation was the mandatory use of masks by all professionals in the centres, but not users.

The centres remained completely closed to visitors until the end of May 2020. If a person had to enter, they were subjected to 14 days of isolation to prevent possible contagion.

After this date, weekly visits lasting half an hour were authorized in an isolated space in the institution, always with the same person, with a minimum physical distance of 2 metres to avoid any physical contact and supervised by a care worker. In December of that same year, the option of weekly walks was added, first lasting half an hour and increasing to one hour. Upon returning from a walk, the client had to change all their clothing. These more permissive measures were suspended during the various outbreaks of the pandemic. If someone in an isolated group became ill, the other members were also isolated.

With the administration of vaccines, infections decreased, and restrictive measures were relaxed.

In June 2022, the health authorities decided that persons with IDD were not a risk group for COVID-19, and consequently care workers were allowed to work without a mask while clients were allowed to return to the lifestyle they had before the pandemic (Bioethics Committee of Catalonia, 2023).

For persons with IDD, the stopping of routines, the difficulty in understanding the situation, and the inability to enjoy specifically chosen, meaningful activity during lockdown has affected their mental health with greater incidence than the rest of the population, generating disorders, increasing states of sadness, apathy, and low self-esteem, and bringing about the appearance of challenging behaviours (Courtenay & Perera, 2020). The COVID-19 crisis has had a negative impact on those who use residential resources. These individuals had already presented with high levels of stress and exhaustion prior to the pandemic, which then further aggravated their situation (Cáceres et al., 2022; Patton et al., 2018; Willner et al., 2020).

A key question has driven the present research project: how did the COVID-19 pandemic affect the family members of institutionalized persons with IDD and change their relationships to their loved ones?

The objective of the present study was to examine and understand the experiences of the family members of persons with IDD living in residential care in Catalonia during the period of maximum restrictions of the COVID-19 pandemic.

## 2. Methods

The study did not involve quantitative methodology because official data on institutionalized persons with IDD during the pandemic was aggregated with that of elderly people who had a very different health and demographic profile.

A qualitative methodology was selected because despite not having reliable quantitative data, it was perceived that morbidity and mortality was not comparable to that of the elderly population and that sharing the same protective measures would have repercussions on institutionalized people and, consequently, on their families.

A qualitative phenomenological interpretative approach was used following the recommendations of qualitative research in health care (Pope et al., 2000). The study was approved by the research ethics committee of the University of Barcelona the coordinating body of the project, with Institutional Review Board number IRB00003099: 21 February 2022.

### 2.1. Participants and setting

All IDD centres where participants were selected form part of the public health system, although most belong to private non-profit foundations.

To identify and enrol participants, purposive sampling was carried out, thus improving the quality standards of the study (Campbell et al., 2020). Variability in terms of age, familial relationship, gender, place of residence and time spent in the centre was considered. Participants were selected by the personnel at the institutions. Data collection was finalized upon reaching theoretical saturation of data (see Table I).

**Table I.** Participant characteristics.

Parentage/rol	Age	Ocupation	Age/gender resident	Institution	Years in the institution	Diagnosis	Place during the pandemic
P1- Mother	86	Retired	48. Woman	Residential	23 years	Down’s Syndrome	Institution
P2- Mother	67	Administrative	42. Woman	Supervised group suport	12 years	Limited mental retardation	Institution
P3-Parents*	83/80	Retired	55.Man	Residential	30 years	Autism	Institution
P4-Mother	72	Retired	42.Woman	Residential	11 years	No diagnosi	Institution
P5-Mother	55	Social health assistant	21.Woman	Supervised group suport	2 years	Rett Syndrome	Home
P6-Parents*	75/71	Retired	46.Man	Supervised group suport	26 years	Frontal Syndrome	Home
P7-Mother	69	Retired	38.Woman	Supervised group suport	5 years	Child brain paralysis	Home
P8-Parents*	60/63	Social health assistant	38. Woman	Residential	3 years	Child brain paralysis	Home
P9-Sister	59	Bussineswoman	57. Woman	Supervised group suport	11 years	Autism	Home
P10-Sister	62	Pedagogue	67. Man	Residential	35 years	Child brain paralysis	Institution
P11-Sister	61	Administrative	64. Man	Residential	40 years	No diagnosis	Institution

*Two parents of de same user.

### 2.2. Measures

#### 2.2.1. Data collection

Data collection was carried out in face-to-face individual interviews that were recorded together with the observations of two researchers between February and October 2022. These dates were chosen for the interviews because although the strictest measures for the centres had not been in place for a few months, there had still been some restrictions in place, such as the use of masks by professionals and avoiding gathering several family members all at once at the entrance of the centres. The aim was for the dialogue to provoke memories of the first days of the pandemic, producing the least possible emotional impact.

Families of clients of the two existing residential care models in Spain (supervised group homes and residential care homes) were selected.

A dialogue technique (Valles, 2009) was used in semi-structured interviews with guiding questions that arose from a discussion group with family members who did not participate in the subsequent interviews, but who described what had the greatest impact on their lives. These findings served as a guide for the study interviews. Interviews were conducted in the dining rooms or rest areas of the centres, with field journals utilized by the researchers. The duration of each interview was approximately 60 minutes. Data collection scheduling was adjusted during the waves of COVID-19 that occurred during the research period.

#### 2.2.2. Data analysis

Data were obtained and studied through interpretive phenomenological analysis (Smith & Fieldsend, 2021). Using ATLAS.ti software, the first two authors reviewed the transcripts independently until they became familiar with the data. First, the characteristics of the IDD participants and their family members were described. Researchers listened to the interviews and read the transcripts to identify and record the predominant themes that emerged from the data. Scientific rigour criteria were considered (Lincoln & Guba, 1985). To achieve reliability, data were discussed among the researchers on the team. To ensure dependability, researchers verified that all identified themes emerged from the inputs of the participants. Purposeful sampling, as well as discussion in all phases of the study, ensured transferability. To achieve dependability, a coding-decoding strategy was used. To ensure confirmability, diversity in terms of age, gender and level of kinship in the participants was established.

Participants were shown their transcripts to ensure no corrections were required. Triangulation was implemented through different perspectives taken from various sources of information.

## 3. Results

### 3.1. Characteristics of the participants

Fourteen relatives of 11 persons living in 10 residential centres in Catalonia, 6 in residential care home and 5 in supervised group home apartments, were interviewed. In most of the cases, only the mother of the resident was interviewed individually. In 3 cases, both parents participated. In another 3 cases, the study participants were sisters who were legal guardians because the parents had died, and these residents were the oldest profile involved in the study.

The average duration of institutionalization for the persons with IDD whose family members were interviewed was 17.5 years, with the shortest duration being 6 months and the longest 40 years. This dispersion is due to the fact that when these persons enter residential care they tend to remain there throughout their lives, and therefore the older their age, the longer the duration of institutionalization.

Persons who lived in group home apartments with support had partial independence for their activities, while those who lived in residential care needed support for almost all of their basic needs. In both types of residence, some users also presented behavioural disorders.

In the study group, there were families who chose not to move their family member from the centre and others who moved their family member to their own home during the period of highest mortality of the pandemic, with stays ranging from 3 to 6 months. After this period, they returned to their residential centre and complied with the health regulations of the time.

In both types of residential centres, the restrictive measures used in elderly care homes were applied.

Table I shows the characteristics of the family members who participated in the study.

### 3.2. Main themes

Our analysis identified 4 main themes that developed into additional themes (Table II).

**Table II.** Main and additional themes.

Main themes	Additional themes
The decision to come home or stay in the residence centre	The centre is safer They will be better protected Home is safer We have space for them We are aging They cannot be at home like this
Fear	Contagion from a relative Contagion of a family member How the life change will affect them
Illness	In the residence, everyone spreads the illness At home, no one spreads the illness Bones also got broken Without prevention
Protocols	Total closure Isolation Distance Communication

#### 3.2.1. Deciding between living at home or in the residence center

At the beginning of the state of alarm in Spain, the management personnel of the residence centres asked family members to decide whether their relatives with IDD would spend the lockdown in the centre or at home, considering that in either case they could not go outside and that if they chose the residence, they would not be able to visit. This decision would remain in place throughout this period.

Family members took the decision based on various factors: the characteristics of the resident and their family, the characteristics of the home and the residential centre, and the possible consequences of each choice.

Some families felt that a private home could better guarantee an avoidance of contagion.

P7: “In the centre, the workers come and go, and they are young people with a more active private life than we do. Since we didn’t have to go to work anymore and were very vigilant, we felt she would be better off at home.” *Mother aged 69 years*

Other families, however, thought that the residential centre, which for them was similar to a health centre and in most cases had large outdoor spaces, was the safest place to stay during the long period of the pandemic.

P11: “We told ourselves that they could run outside, that they would have the forest to go out into and spend their energy, and that they would be alone with only the caregivers wearing masks. They know better than we do. At home they wouldn’t be as well protected as in the centre, so they stayed there.” *Sister aged 61 years*

Although the facilities of the group homes and residential care homes are different, family members were comparing them with their own private homes in terms of comfort. Thus, the stories of the interviewees are similar in both cases.

Another factor in making the decision was on the type of care that the person with IDD would receive in case of infection.

P3: “We thought there would be no better place than the residence if they caught COVID. There they would be well taken care of, and if they had to be hospitalized, the centre would have better contacts than we did to ensure that they would be treated correctly.” *Parents aged 83 and 80 years*

Others felt that in case of infection, the measures taken would have consequences on the person with disability.

P7: “We decided to keep them at home because we could not bear the idea of them having symptoms and being left alone in a room, isolated, and that people dressed as astronauts would tend to them.” *Mother aged 69 years*

For those who decided to take their family member out of the residential centre, having enough space in the home was a determining factor.

P5: “I never appreciated the small space behind the house until COVID happened.” *Mother aged 55 years*

Although they were concerned about cohabitating, these family members felt they had made the right decision because it allowed them to strengthen their relationships.

P9: “If this were to happen again, I would ask for her to come home again. I would sign off right now so that it would continue the same way: have her at home, and we stay home too, because we were working from there.” *Sister aged 59 years*

The family members of residents who presented behavioural problems decided on them remaining in the residence during the pandemic.

P4: “We had no choice, there are two of them and sometimes they are difficult to handle, and we are getting old. With a heavy heart, we decided that they would stay in the residence.” *Mother aged 72 years*

The age of the family members was another factor that impacted the decision.

P1: “I am already quite old. Going to eat with them on the weekend was something I could so, but more time than that is no longer possible, and I couldn’t entertain them more than at the residence either.” *Mother aged 86 years*

All the family members who were interviewed felt their decision was correct, whether they opted for the residential centre or chose to bring them home.

##### 3.2.1.1. Fear

During the first year of the pandemic, the world experienced a situation of constant, collective fear. The family members of persons with IDD experienced different types of fear: fear of contagion, fear of abrupt changes to daily life, and also fear of the vaccine.

These feels were most acute at the beginning of the pandemic, and this was exacerbated by the news emerging from elderly care homes. These feelings were experienced in both family members who chose to keep their loved one in the residence and those who brought them home.

P9: “At the beginning, we were afraid of everything, it seemed like the ‘monster’ was coming to get you in your own home.” *Sister aged 59 years*

Staying at home also meant for the family members that they were afraid for their own health, given that they were the only caregivers.

P6: “We were very concerned about getting COVID ourselves, and especially concerned about him in that case. If we were to get it, who would take care of him?” *Parents aged 75 and 71*

They also reported fear for other vulnerable persons in their home.

P8: “We felt a lot of fear and concern. I worked in elderly care homes without protective gear, and I had my mother-in-law and daughter at home. I could have been a transmitter of the virus. In the end, I was more afraid of having her at home than at the residential center.” *Mother aged 60 years*

Another source of fear that emerged in family members of persons with IDD who were older was the idea of being unable to ensure a dignified, high-quality end of life.

P10: “We always said that if his health were to deteriorate, we would want him to be at home when he died. We were very afraid that if he caught COVID in the residence home, he would die alone. We were so worried that he would feel abandoned!” *Sister aged 62 years*

Family members also expressed concern for the decrease in expressive, emotional and physical abilities due to the lack of routines that had been followed at the centres.

P5: “It was very difficult to manage. She lost her routines and social contact. She didn’t understand anything … ” *Mother aged 55 years*

When vaccines were administered to care home residents in March 2021, an additional fear of adverse effects emerged.

P8: “We were also concerned about the vaccine. They were the first ones to receive it and it all happened very quickly.” *Father aged 65 years*

#### 3.2.2. Illness

The incidence and prevalence of illness due to coronavirus in persons with IDD who stayed in residential homes is unknown because they were recorded together with elderly residents.

All interview participants who chose to leave their relatives in the residential centres reported that their loved one had been ill with coronavirus.

P4: “The center closed on March 13, and my daughters caught COVID at the end of March. We were extremely concerned; nothing was known about the virus. People were dying and they were there locked up, despite needing to move about … ” *Mother aged 72 years*

Most of the residents who got sick had mild symptoms and stayed in the centre. Only one mother reported that her son became ill in the early days of the pandemic and that he was admitted to hospital with the drastic isolation measures that were taken in that period.

P6: “He was sick with COVID and isolated for a long time, and at that same time we heard that so many were dying … although he was very well cared for, but isolated, alone in his room … ” *Mother aged 71 years*

#### 3.2.3. In addition, some residents presented other health problems that also required hospital care and were affected by restrictions due to the coronavirus.

P11: “He fell and broke his ankle and the doctor told us it was the worst kind of fracture there was. He had to have surgery. It went well. We had good people, people who understood us and also those who treated us as if he had had no issues.” *Sister aged 61 years*

Due to the pandemic, some of the usual check-ups on underlying disease as well as preventive examinations were stopped.

P7: “I would take her to the dentist every four months. With the pandemic, she stopped going for a year, and when she was finally able to go, they had to anesthetize her to extract two teeth because it was too complicated to do it in the dentist’s chair.” *Mother aged 69 years*

#### 3.2.4. Protocols

Prior to the pandemic, few family members of persons with IDD were familiar with the concept of protocols in their daily lives. Starting in March 2020, protocols were used to mediate their relationship with hospitalized relatives.

Protocols varied on a nearly daily basis and were poorly adapted to persons with IDD given that they all had an impact on what these individuals needed most: closeness and contact.

##### 3.2.4.1. Full closure of residences.

On 15 March 2020, the government decreed a state of alarm due to the COVID-19 pandemic, resulting in the total closure of the residences. Thus began a potentially long period of separation between persons with IDD in residential centres and their families, except in cases in which it was decided that the disabled person would go home without additional care support.

For all the family members of persons with IDD, it was very painful because of the length of this period.

P10: “We said to ourselves, for him, the residence is home. Seven months passed before we could hug each other.” *Sister aged 62 years*

P7: “Until Saint John’s Day (June 24), we agreed to have her at home, without questioning anything. From then on, it was a struggle to normalize the situation. It was unfair that in order to return to the centre she would be isolated for 15 days, even without presenting symptoms and with a negative PCR result. She returned to the centre in October, when they changed the protocol.” *Mother aged 69 years*

#### 3.2.5. Isolation

Family members were prohibited from entering the residential centres of persons with IDD from March to June 2020.

P2: “They were all locked up, they were confined, to protect the residents. She was used to another dynamic, going out, coming home, so it was all very different.” *Mother aged 67 years*

When residents were later able to go to the family home, upon returning to the centre they had to follow strict hygiene rules and take a PCR or rapid antigen test that required a negative result. All the measures, and these ones in particular, were very complicated to carry out.

P8: “We could have had her at home more often, but every time she came out there was a PCR test and for her it was excruciating, so we decided to see her less.” *Mother 60 aged years*

#### 3.2.6. Distance

In June, when the general population resumed normal life, persons with IDD in residential centres continued to be unable to leave the centre. They were allowed to receive one weekly visit from one relative who was always the same person, maintaining 1.5 metres.

P1: “I have him in front of me and there he is, poor thing, sitting there and me here. What kind of rule was this? We couldn’t even shake hands. He accepted everything.” *Mother aged 86 years*

P10: “We could finally see each other! With a table between us and a person there supervising: Frederic, here I am!” *Sister aged 62 years*

Some residents were unable to cope with the process at all.

P4: “The visits? They were an abomination, not able to touch each other, not understanding anything, jumping on top of the table. And afterwards we couldn’t go to the nearby park. If the residence had had a garden, we would have been able to go outside.” *Mother aged 72 years*

Weekly 30-minute walks with family members in the open air, without physical contact, were introduced seven months after the start of the lockdown.

P5: “A dog had more freedom than we did.” *Mother aged 55 years*

#### 3.2.7. Communication

Communication was one of the areas with the most incidents during the COVID-19 pandemic.

Contact by telephone was used between sick relatives and their families. In some centres, email was also used, and WhatsApp groups that included all the families were created. The latter channel was used to share general information such as changes in protocols and restrictions.

P4: “And when we could finally go outside with them, boom, someone was infected. Change of protocol! Locked down again.” *Mother aged 72 years*

Due to their age, the family members who were interviewed were not native to communication technologies, and therefore needed to learn to socialize using these tools; for several months, online communication was the only option. This solution was positively rated because information was sent much more quickly than by telephone. However, the majority of persons with IDD were unable to understand how to use it.

P8: “We could see her, but she didn’t look at us.” *Mother aged 69 years*

P11: ”‘Why are you inside a telephone?’ my brother said to me.” *Sister aged 61 years*

In addition to high technology, old-fashioned solutions were also used.

P2: “The balcony, on the first floor high up. I would be on the street level and she on the balcony, and we would shout our conversations at each other.” *Mother aged 67 years*

Family members were aware of the contradictions that arose in these protocols, which were very restrictive in the centres and permissive when the same individuals did other activities.

P1: “When he went to the leisure center on Saturdays, they let him do all kinds of sports, and yet there in the residence they could not do any at all.” *Mother aged 86 years*

Most family members agreed on the nearly complete lack of presence of persons with IDD in the media. These feelings were aggravated by the way they were assimilated with the community most poorly treated in the pandemic, residents of elderly care homes, something that exacerbated their fears.

P4: “Everything was about elderly care homes. Were ours just the same? We didn’t appear anywhere.” *Mother aged 72 years*

Family members reported that the design of the protocols did not take into consideration the needs of the persons with IDD and thus reflected a disregard for this group.

P10: “The measures were very drastic in order to be protective, but no one thought about the families. They used poor criteria for this type of residence and did not take care of the emotional aspects.” *Sister aged 62 years*

P5: “We suffered a lot because of the protocols. No one talked about us ever. If a disabled person died, it didn’t affect anyone but the family.” *Mother aged 55 years*

## 4. Discussion

Like other works on persons with IDD, the present study shows that most care givers of vulnerable persons are women (Arora et al., 2020). In the case of IDD, mothers are the most common, and when they are not present, they are substituted by sisters (Oñate & Calvete, 2017).

At the beginning of the pandemic, family members had to choose where to house their loved one. Some studies have described how staying home was a cause of frustration and anxiety, causing an increase in stress and challenging behaviour in persons with IDD (Gacek & Krzywoszanski, 2021; Schuengel et al., 2020). In the present study, this was not the case as the persons with behavioural disorders remained in the residence centres. The psychological strain experienced by both the residents and their families was due to fear of the disease and death, in addition to the effects of isolation (Brooks et al., 2020; Lund et al., 2020; Oomen et al., 2021).

As in other studies, the families who were interviewed highlighted the importance of socialization and maintaining routines in persons with IDD (Paulauskaite et al., 2021), and the stopping of daily routines and therapies during quarantine contributed to increased physical and mental health deterioration in residents (Courtenay & Perera, 2020; Kim et al., 2021; Theis et al., 2021). In our study, all individuals who fell ill with COVID-19 were infected at the residence, a finding that correlates with those of other authors, in which there were few cases of contagion in private homes (Navas et al., 2020).

Our study participants, as in other studies, perceived measures to be disproportionate, difficult to carry out, and incomprehensible to residents and their families (Cáceres et al., 2022). The pandemic has brought to light the fragmentation that exists between social services and health services, with the former having been given the responsibilities and functions of the latter (WHO, 2020) at the start of the pandemic with inadequate protection measures within a context that brought about deficits in material and human resources (Navas et al., 2020). The United Nations has reported that not only the pandemic but also the measures applied to combat it have threatened the rights of persons with IDD (United Nations, 2020), as the study interviewees have also indicated. At the same time, the European Association of Service Providers for Persons with Disabilities (EASPD, 2021) has stressed that the specific needs of this population group were not considered in the planning of the measures that were adopted during the COVID-19 pandemic. In this line, the participants of the present study reported that the measures applied to family members were designed for elderly residents and did not meet their needs, an observation that was also reported in a previous study (Dickinson & Yates, 2020).

On the other hand, families described how the measures proposed to contain COVID-19 in residential centres was totally incomprehensible for persons with IDD, an opinion found in previous work (Courtenay, 2020; Embregts et al., 2022; Samboma, 2021). Such measures undermined communication, with residents finding themselves with people whose faces they could not see at a distance, and above all, deprived of contact with their families (Courtenay & Perera, 2020). This was one of the reasons why some residents were taken to live at home. Communication between residents and their families was mostly by video call, although given the communicative difficulties of the residents, this was not rated especially well by the study participants (Navas et al., 2020).

Family members reported that in their view the measures proposed for residential centres for people with IDD were designed to stop infection, and therefore mortality, in elderly nursing homes. Such measures did not consider the needs of people whose daily lives are affected by human contact that is physical, visual and emotional, something that was truncated with the measures taken in the pandemic (Thurman, 2009).

### 4.1. Study limitations

No studies have been found that focus on persons with IDD who require general support as in the present study, and therefore some of the results have not been compared with previous research. The interviewees who were selected did not lose their resident family member to COVID-19. This selection was made, on one hand, to avoid having these family members relive their loss, and on the other hand, to determine what were the perceptions of most family members. The findings cannot be generalized to other communities or other countries. However, even if they cannot be extrapolated, they can meet the conditions of transferability.

## 5. Conclusions

The family members of persons with IDD took the decision to bring their loved one home or keep them in the residence by considering the dimensions of the home, the behaviour of the resident, and the age of the care takers. None of the interviewees reported that they regretted their decision.

For some families, the residence centre was viewed as a protective place, while for others it was a risk factor for contagion.

Fear of contagion was added to the fear of decline in capacities and the loss of emotional ties.

In all families who participated in the study, the persons with IDD who became ill were the same individuals who remained in the residence centre.

Online communication, while positively rated by the family members, was also a source of unease and confusion for the residents.

Those who had lived in the institution for the greatest number of years were reported to have had difficulties in adapting to living at home again.

All interviewees considered the protocols to be inadequate and unfit for the needs of the residents because they were unable to understand and comply with them.

The family members of persons with IDD who live in residential centres believe that this community is of no importance to the authorities. The measures were disproportionate, and their needs were not considered, given that the recommendations were aimed at elderly care homes.

### 5.1. Short biographical

#### 5.1.1. Montserrat Puig Llobet

RN, Bd, PhD, professor, Department of Public Health, Mental Health and Maternal-Child Nursing, School of Nursing, University of Barcelona, Direction of 8 doctoral theses in the line of positive mental health in different populations and in different doctoral programmes. Tutor of 8 doctoral theses. 10 theses read. Participation as principal investigator in 3 competitive research projects and 9 in non-competitive projects. Participation as collaborator investigator of 6 national, 2 regional and 2 international competitive projects. Evaluator in evaluation agencies and scientific reviewer in journals indexed in JCR. Author of more than 100 articles, 55 indexed in JCR.

### 5.2. Montserrat Roca Roger

RN, Bd, PhD, retired ProfessorDirector and tutor of different doctoral theses. Participation as principal investigator and collaborator investigator in different research projects. Author of various articles and book chapters.

### 5.3. Teresa Nicolàs Silva

RN, residence i CAE for People with Intellectual Disabilities La Vinyota Participation as collaborator investigator in a 1 competitive project. author of various articles, communications, and book chapters.R

### 5.4. Gemma Pérez Gimenez

RN, MSc.PhD,head of Sick Medical Nursing Area and alternatives to hospitalization. Granollers General Hospital, Granollers. PhD in Associate professor in Tecnocampus Participation as collaborator investigator in different competitive project. Author of various communications and book chapters.

### 5.5. Zaida Agüera Imbernon

RN Bd PhD, lecturer in the Department of Public Health Nursing, Maternal and Child Health and Mental Health at the University of Barcelona. Degree in Psychology (2005), with a Master’s Degree in Clinical Psychology (2007) and Neuropsychology (2020). PhD from the UB since 2014. Scientific career with more than 100 publications in JCR and participation in national and international research projects. External evaluator of ANEP since 2017. Training capacity guaranteed by tutoring 10 final degree projects, 10 final Master’s projects and the co-direction of 5 doctoral theses, 4 in preparation and one defended in 2021 with Cum Laude qualification.

### 5.6. Maria Teresa Lluch Canut

RN Bd PhD. professor of Psychosocial Nursing and Mental Health at the University of Barcelona. Principal investigator in 6 competitive projects and collaborator in 20 competitive projects. More than 80 publications indexed in JCR, more than 20 in relevant authorship position. He has directed 30 doctoral theses and another 5 in progress in different doctoral programmes. External evaluator of research projects. Evaluator of scholarships and financial aid in different organizations.

### 5.7. Juan Roldán Merino

RN MSc PhD, head of Studies of the Campus Docent Sant Joan de Déu, a centre attached to the University of Barcelona since 2021 and hired as a professor since 2007. I teach Nursing Degree, official and professional Masters of the UB., Co-direction of 15 doctoral theses. Of which 3 in the line of positive mental health. I am currently co-directing 10 doctoral theses. Regarding research, my scientific production consists of 70 international publications indexed in the Journal Citation Report and more than 20 publications indexed in other databases.

### 5.8. Carmen Moreno Arroyo

RN MSc. PhD, professor of the Department of Fundamental and Medical-Surgical Nursing of the University of Barcelona. PhD in Nursing Sciences. 62 articles published (15 in JCR-indexed journals − 4 Q1; 4 Q2; 4 Q3; 3 Q4; 36 in Scopus-indexed journals; and 11 in non-indexed journals). Participation in 33 research projects (13 financed in competitive calls; Member EI). Director of 5 doctoral theses. Member of 9 theses committees. 8 years as an expert evaluator of research projects (Official College of Nursing of Barcelona).

### 5.9. Marta Prats Arimon

RN Bd PhD, associate Professor at the University of Barcelona and PhD from the International University of Catalonia. Professor of different Masters of the University of Barcelona. Participation as principal investigator of 1 competitive and 2 non-competitive research projects. Participation as collaborator investigator of 1 competitive national and 1 regional projects. Scientific reviewer in Health and Social Community journal indexed in JCR, Author of 5 publications, 2 indexed in JCR.

### 5.10. Aurelia Sánchez Ortega

RN Bd PhD, professor at the University School of Nursing and Occupational Therapy of Terrassa. For 15 years I have combined university teaching for Diplomas, Degrees and Masters with the assistance and management of primary care teams. I am currently directing 2 doctoral theses in the line of positive mental health in the doctoral programme of the UB. I have participated in 3 competitive projects as collaborator investigator. I am an evaluator of scholarships and financial aid in different organizations.

### 5.11. Xavier Domènech Mascaró

Bd, MSc, administrative at the Catalan Health Institute. Doctoral student in medicine Bachelor of Business Administration. Diploma in business Master in direction and health management. Participation as collaborator investigator in a 1 competitive project. Author of several communications.

### 5.12. Miguel Angel Hidalgo Blanco

RN MSc. PhD, professor of the Department of Fundamental and Medical-Surgical Nursing of the University of Barcelona. PhD in Nursing Sciences. Author of several articles published in JCR-indexed journals and Scopus-indexed journals. Participation in 6 research projects competitive. Director of 1 doctoral thesis.

### 5.13. Antonio Moreno Poyato

RN MSc PhD,professor of the Department of Public Health Nursing, Mental Health and Maternal-Child Health at the University of Barcelona. Principal investigator in 5 competitive projects and collaborator in 8 competitive projects. More than 35 publications indexed in JCR, more than 20 in authorship position Page 13 of 15 relevant. Directing 15 doctoral theses in progress in different doctoral programmes. External evaluator of research projects. Reviewer of national and international scientific journals indexed in JCR.

## Supplementary Material

SHORT BIOGRAPHICAL.docx
