# Supplementary material for: The experiences of family members of persons with intellectual disabilities who used residential care homes during the COVID-19 pandemic
Source: Int J Qual Stud Health Well-being. 2023 Dec 6;19(1):2288100. doi: 10.1080/17482631.2023.2288100 (PMC11737831; doi:10.1080/17482631.2023.2288100)
Supplement: SHORT BIOGRAPHICAL.docx [file ZQHW_A_2288100_SM6583.docx]

**SHORT BIOGRAPHICAL**

**Montserrat Puig Llobet**

Professor and PhD in Sociology from the University of Barcelona. Nurse and Anthropology. Direction of 8 doctoral theses in the line of positive mental health in different populations and in different doctoral programs. Tutor of 8 doctoral theses. 10 theses read. Participation as principal investigator in 3 competitive research projects and 9 in non-competitive projects. Participation as collaborator investigator of 6 national, 2 regional and 2 international competitive projects. Member of the research team of the SGR 'Nursing care in mental health, psychosocial and complexity' recognized by AGAUR. Evaluator in evaluation agencies and scientific reviewer in journals indexed in JCR. Author of more than 100 articles, 55 indexed in JCR.

**Montserrat Roca Roger**

Retired Professor. Nurse, philologist and PhD in Social Sciences and Health. Director and tutor of different doctoral theses. Participation as principal investigator and collaborator investigator in different research projects. Author of various articles and book chapters.

**Teresa Nicolàs Silva**

Nurse. Residence i CAE for People with Intellectual Disabilities La Vinyota. Mollet Health Foundation. Participation as collaborator investigator in a 1 competitive project. author of various articles, communications, and book chapters.

**Gemma Pérez Gimenez**

Nurse and Head of Sick Medical Nursing Area and alternatives to hospitalization. Granollers General Hospital, Granollers. PhD in Associate professor in Tecnocampus Participation as collaborator investigator in different competitive project. Author of various communications and book chpaters.

**Zaida Agüera Imbernon**

Lecturer in the Department of Public Health Nursing, Maternal and Child Health and Mental Health at the University of Barcelona. Degree in Psychology (2005), with a Master's Degree in Clinical Psychology (2007) and Neuropsychology (2020). PhD from the UB since 2014. Scientific career with more than 100 publications in JCR and participation in national and international research projects. External evaluator of ANEP since 2017. Training capacity guaranteed by tutoring 10 final degree projects, 10 final Master's projects and the co-direction of 5 doctoral theses, 4 in preparation and one defended in 2021 with Cum Laude qualification.

**Maria Teresa Lluch Canut**

PhD in Psychology. Graduated in Psychology. Diploma in Nursing. Professor of Psychosocial Nursing and Mental Health at the University of Barcelona. Principal investigator in 6 competitive projects and collaborator in 20 competitive projects. More than 80 publications indexed in JCR, more than 20 in relevant authorship position. He has directed 30 doctoral theses and another 5 in progress in different doctoral programs. External evaluator of research projects. Evaluator of scholarships and financial aid in different organizations.

**Juan Roldán Merino**

Head of Studies of the Campus Docent Sant Joan de Déu, a center attached to the University of Barcelona since 2021 and hired as a professor since 2007. I teach Nursing Degree, official and professional Masters of the UB., Co-direction of 15 doctoral theses. Of which 3 in the line of positive mental health. I am currently co-directing 10 doctoral theses. Regarding research, my scientific production consists of 70 international publications indexed in the Journal Citation Report and more than 20 publications indexed in other databases.

**Carmen Moreno Arroyo**

Professor of the Department of Fundamental and Medical-Surgical Nursing of the University of Barcelona. PhD in Nursing Sciences. 62 articles published (15 in JCR-indexed journals - 4 Q1; 4 Q2; 4 Q3; 3 Q4; 36 in Scopus-indexed journals; and 11 in non-indexed journals). Participation in 33 research projects (13 financed in competitive calls; Member EI). Director of 5 doctoral theses. Member of 9 theses committees. 8 years as an expert evaluator of research projects (Official College of Nursing of Barcelona).

**Marta Prats Arimon**

Associate Professor at the University of Barcelona and PhD from the International University of Catalonia. Professor of different Masters of the University of Barcelona. Participation as principal investigator of 1 competitive and 2 non-competitive research projects. Participation as collaborator investigator of 1 competitive national and 1 regional projects. Scientific reviewer in Health and Social Community journal indexed in JCR, Author of 5 publications, 2 indexed in JCR.

**M. Aurelia Sánchez Ortega**

PhD in Nursing Sciences, I read my doctoral thesis in 2015, with excellent Cum Laude. Professor at the University School of Nursing and Occupational Therapy of Terrassa. For 15 years I have combined university teaching for Diplomas, Degrees and Masters with the assistance and management of primary care teams. I am currently directing 2 doctoral theses in the line of positive mental health in the doctoral program of the UB. I have participated in 3 competitive projects as collaborator investigator. I am an evaluator of scholarships and financial aid in different organizations.

**Xavier Domènech Mascaró**

Administrative at the catalan health institute. Doctoral student in medicine Bachelor of Business Administration. diploma in business Master in direction and health management. Participation as collaborator investigator in a 1 competitive project. author of several communications.

**Miguel Angel Hidalgo Blanco**

Professor of the Department of Fundamental and Medical-Surgical Nursing of the University of Barcelona. PhD in Nursing Sciences. Author of several articles published in JCR-indexed journals and Scopus-indexed journals. Participation in 6 research projects competitive. Director of 1 doctoral thesis.

**Antonio Moreno Poyato**

Professor of the Department of Public Health Nursing, Mental Health and Maternal-Child Health at the University of Barcelona. Principal investigator in 5 competitive projects and collaborator in 8 competitive projects. More than 35 publications indexed in JCR, more than 20 in authorship position Page 13 of 15 relevant. Directing 15 doctoral theses in progress in different doctoral programs. External evaluator of research projects. Reviewer of national and international scientific journals indexed in JCR.
